# Editorial: Update of Current Evidences in Breast Cancer Surgery

**DOI:** 10.3389/fonc.2022.928467

**Published:** 2022-06-24

**Authors:** Gianluca Franceschini, Lorenzo Scardina, Giuseppe Visconti, Akitatsu Hayashi, Riccardo Masetti

**Affiliations:** ^1^ Center of Multidisciplinary Breast, Agostino Gemelli University Polyclinic (IRCCS), Rome, Italy; ^2^ Fondazione Policlinico Universitario Agostino Gemelli Istituti di Ricovero e Cura a Carattere Scientifico (IRCCS), Department of Women, Children and Public Health Sciences, Breast Unit, Rome, Italy; ^3^ Department of Breast Center, Kameda Medical Center Chiba, Kamogawa, Japan

**Keywords:** breast cancer, surgical treatment, breast-conserving surgery, mastectomy, breast reconstruction, sentinel lymph node biopsy, axillary surgery

Breast cancer is acknowledged as an international priority in health care; it is currently the most common cancer in women worldwide with demographic trends indicating a continuous increase in incidence (Keelan et al.). Significant efforts and resources have been dedicated in order to develop optimal strategies in breast cancer diagnosis and treatment over the last decades; increased population based screening and improved adjuvant therapies have been able to progressively reduce breast cancer mortality rates.

Early diagnosis is increasing in many developed countries thanks to the diffusion of screening programs and improvement of radiological devices despite the negative impact of COVID-19 pandemic (Buonomo et al.) (1).

Nowadays, the therapeutic strategies against breast cancer are increasingly customized for each patient and modulated according to clinical features, staging, biologic factors such as hormone receptor status, Ki67, HER2 overexpression; an accurate discussion with each patient about the advantages and issues associated with the chosen treatment should always be performed in a correct decision-making process.

However, a multidisciplinary management, involving surgical, medical and radiation oncology, is crucial to define optimal strategy, improve oncological and aesthetic results, increase patient’s quality of life and prolong survival (2, 3).

Early-stage breast cancer should usually be treated by primary surgery to the breast and axillary lymph nodes; breast-conserving treatment (breast-conserving surgery (BCS) plus radiotherapy) or mastectomy are the possible surgical options; in both cases, oncological radicality and patient aesthetic satisfaction should always be ensured.

The modern breast surgeon should perform the choice of breast-conserving treatment versus mastectomy based on breast volume to cancer volume ratio, multicentricity, presence of mammographic microcalcifications, ability to achieve clear surgical margins and patient wishes; a careful evaluation of the disease by clinical and radiological examination is crucial to select the optimal local treatment.

BCS combined with adjuvant radiotherapy is now deemed the gold standard approach for early-stage breast cancer because it permits to preserve the breast without affecting oncologic results; various prospective randomized studies have shown no significant differences in disease-free and overall survival rates when comparing breast-conserving treatment with mastectomy for early-stage breast cancer.

BCS should always ensure the complete surgical removal of the tumor with negative surgical margins and an adequate aesthetic outcome followed by adjuvant radiotherapy to eradicate any residual disease. The role of BCS has been also expanded to include some patients who would otherwise require mastectomy to obtain appropriate tumor clearance thanks to the use of oncoplastic techniques (4–6); these innovative procedures combine the principles of surgical oncology and plastic surgery to remove larger amounts of breast tissue with safer margins while improving aesthetic outcomes also with the use of filler biomaterials (7–9).

Mastectomy should be considered when a conservative treatment is unable to ensure appropriate local control and adequate aesthetic outcomes; common indications to mastectomy include extensive or multicentric disease, large cancer size in relation to the breast size that cannot be incorporated by local excision with a satisfactory cosmetic result; persistent positive margins despite multiple re-excisions; inability to perform adjuvant radiotherapy after BCS due to active connective tissue disease involving the skin or previous radiation therapy to the breast or chest wall; presence of BRCA pathogenic variants; patient preference (Yang et al., Li et al.,) (10).

The conservative mastectomies (skin-sparing and nipple-sparing mastectomies) are accepted new techniques that allow to improve aesthetic results and patient quality of life; these mastectomies combine the oncological advantage of the complete glandular excision with the optimal cosmetic result of the conservation of the skin envelope and, wherever possible, the nipple areola complex.

Immediate breast reconstruction with prosthesis or autologous tissue should always be performed after mastectomy as it can enhance the patient quality of life and positively affect their psychological health, sexuality, body image, and self-esteem (Zheng et al.).

Traditionally, reconstruction with prosthesis has been performed by placement of the implant in a submuscular pocket created beneath the pectoralis major muscle; in recent years, the placement of the prosthesis in a prepectoral plane, using polytech prothesis with micropolyurethane foam coated shell surface (microthane), has been increasingly employed (10); prepectoral approach is a safe, reliable and effective alternative to traditional technique while offering better aesthetic outcomes and patient quality life. The increasing demand for further aesthetic result improvement in breast reconstruction after mastectomy has also led to search innovative solutions by endoscopic and robotic approaches in order to limit scar visibility (Lee et al.).

As regards the surgical treatment of the axillary nodes, sentinel lymph node biopsy (SLNB) is deemed the gold standard for nodal staging in patients with early breast cancer and clinically negative nodes (Yoon et al., Wang et al., Xu et al.); axillary dissection remains the standard of care for patients with clinically positive nodes even if new therapeutic strategies are emerging in patients with a pathological positivity in sentinel lymph node (Luo et al., Al-Masri et al.).

Neoadjuvant chemotherapy (NAC) is being used with increasing frequency in the multidisciplinary treatment of patients with operable breast cancer (11). Several clinical trials have proved that NAC permits to achieve essential benefits such as assessment *in vivo* tumor’s chemosensitivity by monitoring response to therapy; downstaging of tumor favoring BCS over mastectomy; reduction of excision volumes in patients with cancer who are already candidates for BCS; downstaging of the axilla in order to avoid complete axillary dissection (Lee et al.,) (12).

The locoregional treatment of metastatic breast cancer is largely reserved for palliation in patients with significant symptoms from primary tumor (13). The efficacy of this surgery is still controversial and the debate about resection of primary tumor in metastatic breast cancer patients persists (Zheng et al., Wang et al.). Surgical treatment of primary breast cancer in metastatic setting could be an option after systemic therapies. Randomized prospective trials for each immunophenotype are necessary in order to confirm this evidence (Zhang et al., Zhou et al.).

In conclusion, this Research Topic offers a set of evidence-based practice articles useful to optimize the surgical treatment of breast cancer patient by a multidisciplinary and personalized approach.

## Author Contributions

Contribution Author(s) Manuscript preparation: LS, GF Manuscript editing: LS, GF Manuscript review: RM, GF. All authors contributed to the article and approved the submitted version.

## Conflict of Interest

The authors declare that the research was conducted in the absence of any commercial or financial relationships that could be construed as a potential conflict of interest.

## Publisher’s Note

All claims expressed in this article are solely those of the authors and do not necessarily represent those of their affiliated organizations, or those of the publisher, the editors and the reviewers. Any product that may be evaluated in this article, or claim that may be made by its manufacturer, is not guaranteed or endorsed by the publisher.
